# “Lock and Protect”: Development of a Digital Decision Aid to Support Lethal Means Counseling in Parents of Suicidal Youth

**DOI:** 10.3389/fpsyt.2021.736236

**Published:** 2021-10-06

**Authors:** Joan R. Asarnow, Lucas Zullo, Stephanie M. Ernestus, Chase W. Venables, David B. Goldston, Angela M. Tunno, Marian E. Betz

**Affiliations:** ^1^Department of Psychiatry and Biobehavioral Sciences, University of California, Los Angeles, Los Angeles, CA, United States; ^2^Department of Psychology, Stonehill College, Easton, MA, United States; ^3^Department of Psychiatry and Behavioral Sciences, Duke University School of Medicine, Durham, NC, United States; ^4^Department of Emergency Medicine, University of Colorado School of Medicine, Aurora, CO, United States

**Keywords:** suicide, firearms, children, adolescents, suicide attempts, self-harm

## Abstract

**Objective:** Reducing access to lethal methods is an effective suicide prevention strategy that is often neglected in routine care. Digital interventions have shown promise for addressing such gaps in care; and decision aids have proven useful for supporting complicated health-related decisions, like those involving lethal means restriction. This article describes a parent/caregiver-facing web-based decision aid, the development process, and user testing.

**Method:** A user-centered, participatory, mixed methods development design was employed. Beginning with an adult-focused decision aid developed by members of our team, we assessed ten iterations of the parent/caregiver decision aid with stakeholders (*N* = 85) using qualitative interviews and quantitative surveys. Stakeholders included: parents/caregivers whose children had histories of suicidal episodes before age 25, young adults with histories of suicidal thoughts/behaviors, firearm owners/representatives from firearm stores/ranges/groups, mental and medical health care providers, and emergency responders.

**Results:** The final “Lock and Protect” decision aid was viewed as “useful for changing access to lethal means” by 100% of participants. Ninety-four percent of participants rated the information on reducing access to lethal means as good to excellent, and 91% rated the information on storage options as good to excellent. Qualitative feedback underscored a preference for offering this digital tool with a “human touch,” as part of safety and discharge planning.

**Conclusions:** “Lock and Protect” is a user-friendly web-based tool with potential for improving rates of lethal means counseling for parents/caregivers of suicidal youth and ultimately reducing pre-mature deaths by suicide.

## Introduction

Suicide is currently the second leading cause of death in adolescents and young adults in the United States (US), and current data indicate that suicide death rates are increasing (1, 2). Availability of suicide attempt methods (e.g., guns, pills, ligatures) increases the likelihood of attempts, and the specific method used affects likelihood of death. Reducing access to lethal methods is an effective suicide prevention strategy (3–6) and goal six of the US National Strategy for Suicide Prevention (2012) (7).

Lethal means counseling often emphasizes firearms, a method with case fatality rates reaching 85–90% (8, 9) and accounting for 41.2% of pediatric suicide deaths (1). Living in a home with firearms is associated with a three- to four-fold increase in adolescents' risk of suicide death, and projections estimate that limiting firearms access among youth at risk for suicide has potential for preventing thousands of deaths each year (10–12). Despite many contact points where lethal means counseling could occur, rates as low as 4% are reported in some emergency departments (EDs), the setting where most youth making medically serious suicide attempts receive care (13–17). Only about a third of EDs describe lethal means counseling as part of routine care after a suicide attempt (18).

Web-based and other types of digital interventions have strong potential for augmenting services and addressing service gaps attributable to time and staffing constraints as well as other barriers (e.g., insufficient training, provider discomfort) that may contribute to low rates of lethal means counseling. The standardization and branching capabilities of a digital tool combined with the ability to confidentially address sensitive topics (e.g., gun ownership) may also enhance intervention acceptability and effectiveness. Digital interventions are also easily scalable for broad dissemination and offer an approach for expanding on face-to-face services.

Decision aids (DAs) can be developed as digital tools that can augment clinician interactions by offering patients education on potentially difficult decisions, considering their values and personal preferences (19–21). These clinical tools have been used to support complicated health-related decisions such as those involving medical treatments, end of life care, and disease screening. Systematic reviews support the benefits of DAs for enhancing decision-making, decreasing decisional conflict, and improving patient-provider communication and shared decision making (22–24). More specifically, a recently developed web-based DA to support lethal means counseling in suicidal adults (“Lock to Live,” L2L) has demonstrated feasibility and acceptability with ED patients and providers (19–21).

Recognizing that children and adolescents are generally in the care of parents or caregivers (hereafter referred to as parents), we developed the first, to our knowledge, DA to support parents in considering and developing options for enhancing their child's safety after a suicidal episode. The DA approach of supporting informed decision-making was viewed as fitting parents' responsibility to protect their children. In this article, we describe our DA development process and final web-based parent-facing DA. Consistent with the Accelerated Creation-to-Sustainment (ACTS) framework for mHealth tool development (25), we (1) employed a user-centered participatory design; and (2) focused on the “create/development phase” with the aim of constructing an initial DA and implementation blueprint for proceeding to the “trial/evaluation phase” (25).

## Methods

### Design

A mixed methods approach that combined qualitative and quantitative data was utilized. Consistent with the International Patient Decision Aids Standards (IPDAS) (26) and Ottawa Decision Support Framework (27), the DA aimed to: support parents in making a decision that is informed by and reflective of their values; provide information to inform decision making; and frame options within a behavior change context, gently “nudging” parents toward behavior change while respecting and supporting parent agency (28).

Our design team included individuals from the adult L2L team and others with expertise/experience with: firearms; suicide and injury prevention; child and adolescent psychology, psychiatry, and mental health; public health; decision science; dissemination and implementation science and practice; and DA design. This team met throughout the study to consider artwork and messaging, user experience, and programming considerations. As in L2L (19), the DA included typical DA content customized for the decision of which options to choose to enhance protection and reduce access to high lethality methods. This included: (1) an introduction specifying the decision; (2) presentation of options, and pros and cons to consider; (3) clarification of preferences, logistics and considerations; and (4) consideration of next steps that would encourage and begin behavior change to enhance youth safety.

### Participants

Stakeholders (e.g., parents with lived experience, firearm owners) were recruited through outreach/advertising to communities with lived experience with suicide and suicide attempts, knowledge of firearms, suicide prevention care, and emergency responders (police/EMS/fire department). Snowball sampling was used, where participants supported recruitment by telling others about the study and how to reach the study team. Participants were recruited nationally, with stakeholders included from a variety of states and regions across the country. Inclusion criteria required that stakeholders fit one or more of the following criteria: parent whose child had a history of suicidal ideation or suicide attempts before age 25; young adult (<25 years) with prior suicidal ideation or suicide attempts; firearm owners or representative from a firearm store/range/group; medical or mental health care provider; and emergency responder. Exclusion criteria were: cannot read English; age <18 years.

### Procedures

Following brief eligibility screening and consent, confidential interviews were conducted by telephone, Zoom, or in person. During the interviews, participants were sent/emailed the current DA version or link to the DA version once programmed. Interviews explored participants' decision-making regarding safe storage and reducing access to suicide attempt methods, recommendations for DA changes, and perceptions regarding effects of the DA on decision-making. Interview duration was roughly 45–90 min for initial iterations and shortened (~20 min) for the final version when the goal was to assess reactions to the final DA. Participants completed online surveys asking about demographic characteristics and DA acceptability (28). The Acceptability Questionnaire used in the online surveys is presented in Supplementary Figure S1. Interviewers completed de-identified reflective notes, covering the content of the interviews, unique reactions to the DA, and any relevant content. Notes were reviewed in team meetings and an audit document summarized feedback and changes made.

We began with the 2019 version of L2L, which focused on firearm safety and was developed through 29 iterative versions and interviews with 64 adults (19). Themes emerging from this adult-focused work included the importance of combining messages of hope with educational information; the importance of developing the DA so that it was concise and also thorough, non-judgmental, trustworthy, and accurate; the need for high acceptability across diverse stakeholder groups, such as firearm owners, clinicians who might use the DA, and family and friends of the patient. We tested L2L for parents, used the collective L2L data to begin developing a DA specifically for parents, and created the “Lock and Protect” (L&P) DA for parents based on testing 10 L&P iterations.

Analysis of findings occurred throughout testing. All identified usability and acceptability problems were considered hypotheses to test with subsequent users. Global concerns generated with early iterations were revisited during subsequent test waves to ensure that problem-solving efforts achieved desired results. Due to diversity in stakeholder perspectives and frequent conflicting feedback, the development team considered feedback and made changes by consensus. The test rounds were stopped based on two criteria: (1) consensus/minimal variance was observed on the rating indicating that the DA was useful for “changing access to lethal means;” and (2) stakeholder feedback during qualitative interviews was judged to be stable, generally consistent, with no new themes emerging from additional participants.

Initial versions of L&P were on paper or pdf to facilitate adjustments. After the eighth iteration, when feedback was deemed consistently positive across stakeholders, a web-based version was developed for testing. This allowed use on tablets and mobile phones. The final L&P iteration had a Flesch-Kincaid Reading Ease Score of 76.9 and 7th grade reading level (29). To develop guidance on integration within workflow, we conducted a testing round with clinicians focusing on optimal strategies for integrating L&P within clinical workflow in EDs and emergency care services. The final test round included parents of youth who had signs of suicide attempt risk (e.g., suicidal ideation or behavior, depression) to confirm that L&P was ready to progress to clinical trial testing. The study protocol was approved by the IRB.

## Results

Between 4/3/18 and 3/18/21 we recruited 85 stakeholders; most (78%) participants were parents, 89% had lived experience with suicidality (in child, family member, self, or acquaintance), 49% owned firearms, and race/ethnicity of most was Non-Hispanic white (see Table 1 for additional description of participant characteristics).

**Table 1:** Demographic characteristics (*N* = 85 stakeholders).

	***n***	**%**
**Parent**	66	77.6%
**Parent of child with history of suicidality**	34	40.0%
**Firearm owner or representatives from firearm store/range/group**	42	49.4%
Individual with lived experience with suicidality^*^	76	89.4%
**Mental health care provider**	21	24.7%
**Medical health care provider**	16	18.8%
**Emergency responder**	12	14.1%
**Age (Mean, SD)**	49.5	14.2
**Sex**		
Female	45	52.9%
Male	40	47.1%
**Race**		
White	68	80.0%
African-American	6	7.1%
Asian	2	2.4%
Native American	1	1.2%
Biracial	2	2.4%
Other	1	1.2%
Not answered	5	5.9%
**Hispanic/Latinx Ethnicity**	8	10.5%
**Veteran status**	5	6.6%

* *Experience with suicidality in: child or self (n = 22, 25.9%); family member (n = 12, 14.1%); and/or knew someone personally who died by suicide (n = 60, 70.6%). Total exceeds N = 85 because stakeholders could indicate the presence of multiple types of lived experience*.

### Development

**Figure 1:**
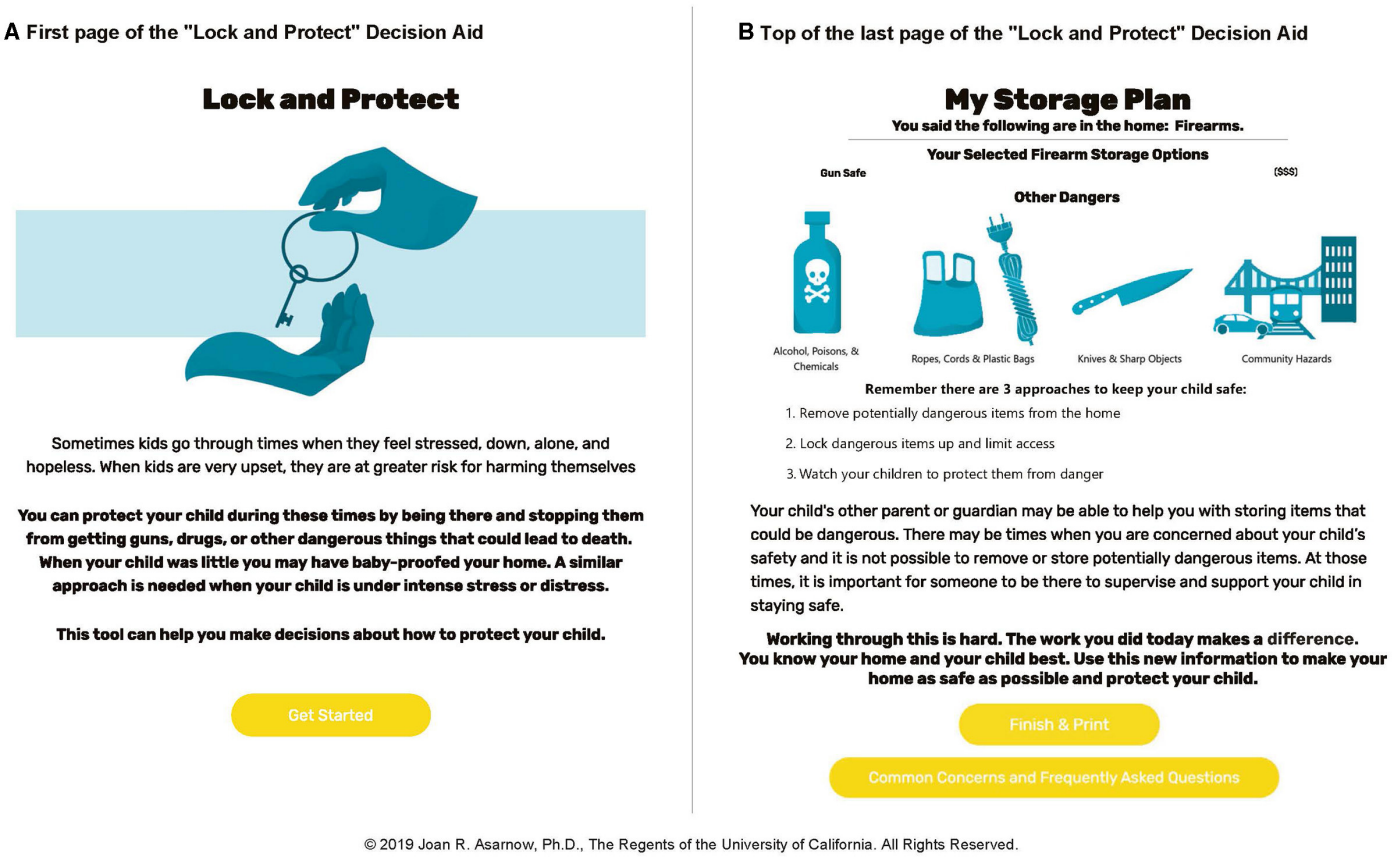
Screenshots from “Lock and Protect” Decision Aid: **(A)** First page of the “Lock and Protect” Decision Aid. **(B)** Top of the last page of the “Lock and Protect” Decision Aid.

L&P was developed through 10 iterative versions (Supplementary Table S1). We began with the L2L DA designed for suicidal adults and tested an introduction focusing on the importance of reducing access to lethal means in children. There was a strong consistent preference for creating a DA specifically designed for parents of youth with elevated suicide risk, leading to development of the L&P DA which initially focused only on firearms (like the original L2L). Based on participant feedback, we expanded the focus to include medicines/poisons and other potentially lethal methods (e.g., methods of hanging and suffocation, jumping off high buildings), as well as protective and supportive supervision/monitoring as a means of preventing youth from completing potentially deadly actions. We adopted the analogy of “child-proofing the home with younger children,” gently nudging parents toward actions to similarly initiate protective steps when their child was at risk (Figure 1A). The shift in title “Lock & Protect” also emerged from this feedback and comparisons with other titles (e.g., Lock to Live, Lock to Protect) and was selected to convey that storage and other protective actions are needed when a child is at risk. Versions 0–10 addressed firearm safety, 5–10 medicine/poison safety, and 9–10 included broader sections on the need for protective and supportive supervision/monitoring. This expanded focus was based on: participant feedback; a desire to have a DA that would be useful across a broader population; recognition that overdose is the most common suicide attempt method in youth; data indicating that suffocation and hanging are major suicide death methods in young people in addition to firearms; and acknowledgment that all deadly methods cannot realistically be eliminated from the environment (7).

Table 2 summarizes key elements in the final iteration of L&P. The DA begins with a brief introduction stressing that firearms and other methods can be highly lethal methods for suicide attempts, the importance of limiting access to potentially lethal methods when a child is at risk, and consideration of who could help the parent store and limit access to potential suicide attempt methods. For respondents indicating that firearms are “in or near the home,” the DA progresses to clarification of values (e.g., role of cost in decisions), followed by presentations of decision options considering variation in choices (temporary storage in the home vs. outside the home), and asks parents to make a decision about what option would work best for their situation based on personal values and considering the pros and cons of different options. To maintain privacy, an issue identified as important particularly as related to firearms, individuals' responses on the DA are not saved. After completing the firearm section, the DA branches to medicine storage.

**Table 2:** Summary of “Lock and Protect” sections, key elements, and goal and rationale for each section.

	**Imagery**	**Messaging and key points**	**Goal and rationale**
Introduction	Hands with key	Stress and distress can lead to self-harm, build hope, compare protective action to child-proofing home when child was little.Identify decision: How to protect your child when at risk of getting guns, drugs, or other dangerous things that could lead to death.	Engage and encourage tool use, nudge toward increased protection.
Background	Youth on bench	Temporary nature of risk states. Rationale for storing potentially dangerous methods of suicide/self-harm	Nudge toward protection through safe storage.
Issues #1	Figure with caring people	You don't have to do this alone. Other(s) can help with safe storage.	Nudge toward identifying others who can help.
Issues #2	9 of 10 people attempting suicide by firearms die	Firearms are the most lethal suicide attempt method. First step is to decide whether your child can access firearms in or near home	Increase recognition that access to firearms can increase risk of suicide death.
Issue #3	Home and storage site	Consider firearm storage options. Two types of temporary storage options: in the home; or outside the home.	Nudge toward safe storage, some firearm owners will not be open to out-of-home options
Issues #4	Price tag	Varying costs for storage optionsConsider importance of cost for you.	Encouraging unrealistic options may be counterproductive
Issues #5	Paper with check	Some options require background checks	Some firearm owners will not be open to background checks
Table of options	Expandable rows	You can choose a temporary storage option that works for you. Displays options, stories of others in similar situations.	Supports informed decision making
Medication introduction	–	Medicines and poisons can be lethal. When child is at risk, consider safe storage of substances.	Nudge toward safe storage when child at risk.
Issue #3	Medicine bottle with pills	Consider needs that that impact storage. Some medicines need to be easily available, some can be removed.	Nudge toward acceptable storage options
Table of options	Expandable rows	You can choose a storage option that works for you. Displays options, stories of others	Supports informed decision making
Other Available and Lethal Methods	Circle of other available lethal methods (e.g., hanging, traffic)	There are other potentially deadly suicide attempt methods that cannot be completely eliminated. Consider risk outside and inside the home and possible protective actions.	Nudge toward protective action through offering multiple strategies, combat feelings of helplessness.
Issue #1	Image of youth with adults	When child is at risk, making sure they are not alone and with someone who can keep them safe is another way to protect. When unsure don't take risks.	Nudge toward an active vs. helpless stance. Decide whether there are times when your child is alone and not monitored.
Table of options	Expandable rows	Emphasize choice, displays options for ensuring that child is not alone and is monitored in a supportive way that will provide protection	Supports consideration of strategies for supportive and protective monitoring and supervision.
Summary	Displays prior selections and dangerous suicide attempt methods	Summarizes and praises effort. Lists choices on storage options, people to help, crisis hotlines, reminds of 3 strategies for protecting child (remove dangerous items, lock up and store dangerous items, watch your child). Option to print summary, provides link to common concerns and frequently asked questions (FAQ)	Provides printable summary for individual to review with provider and take home as reference
Frequently Asked Questions (FAQs)	At top of pages	Responds to common questions and concerns (e.g., laws, how to begin, how to start conversations, suicide methods)	Allows further explanation while keeping text sparse on core pages
Talk to Someone	At top of pages	Help is available. Provides crisis telephone and text numbers, 24/7 access	Provides link to crisis line information on all pages

Respondents indicating no firearm access are taken directly to the medicine section. Following a similar format, parents are asked to consider whether there are medicines, chemicals or poisons in their home. If they respond affirmatively, they are asked to consider their personal situation (e.g., whether family members need daily and emergency access to medicines, presence of expired medicines), are provided with information about different storage options, and asked to make a decision regarding what would work best in their situation. Individuals indicating no access to medicines, chemicals or poisons are branched directly to the next DA section, which highlights other potentially deadly suicide attempt methods that cannot be completely eliminated (e.g., jumping from high buildings, traffic accidents, sharps used for cutting, ropes/ methods used for hanging/suffocation). The message is that, despite challenges, parents can protect their children through (1) limiting access when possible, (2) having a list of emergency response numbers and lifelines available, and (3) having a supportive and responsible adult with the child. Parents are then asked to consider whether their child is ever left alone, and if so, taken to a list of strategies for supportive and protective monitoring (e.g., responsible adult, trusted and responsible peer, pleasant activity where monitoring is present and consistent, monitoring device), as well as limitations and advantages of different approaches.

The final section of L&P is a printable summary page which lists the parent's selections, and reminders of protective strategies (Figure 1B). Crisis hotline information and a link to a separate page on common concerns and FAQs are also available on the summary and at the top of all screens.

### Acceptability of Final “Lock and Protect” Decision Aid

Thirty-three participants (21 parents whose children had past suicidal ideation and/or behavior; 12 clinicians who worked with suicidal and self-harming youth, 9 owned firearms) viewed and rated the final L&P iterations (9 and 10). Table 3 presents details on stakeholder ratings for each item of the Acceptability Questionnaire. Ratings indicated strong acceptability, with 100% of participants rating L&P as “useful for changing access to lethal means,” 94% rating the presentation of the role of lethal means in suicide prevention as good to excellent, 94% rating the presentation of information on lethal means as good to excellent, 91% rating the information on storage options as good to excellent, and 91% indicating they thought L&P presented options in a balanced way. Lower ratings were obtained for DA length (64% just right, 27% too long, 3% too short, 6% unsure). Similarly, 64% thought the amount of information presented was “just right,” while 21 and 15%, respectively thought information was too much or too little. Interestingly, all participants wanting more information were parents with lived experience. Some content areas were not emphasized in order to reduce length which may have contributed to lower ratings on unprioritized areas (e.g., types of research studies) as shown in Table 3.

**Table 3:** Stakeholder ratings of the final version of the “Lock and Protect” decision aid on the acceptability questionnaire (*N* = 33).

	**Yes, f (%)**	**No, f (%)**			**Skip/unsure, f (%)**
Useful for changing access to lethal means	33 (100%)	0 (0%)			0 (0%)
Quality of presentation rated by content area^*^	**Poor, f (%)**	**Fair, f (%)**	**Good, f (%)**	**Excellent, f (%)**	**Not rated, f (%)**
Role of lethal means (e.g., firearms, medications)	0 (0.0%)	2 (6.1%)	14 (42.4%)	17 (51.5%)	0 (0.0%)
Options for reducing access to lethal means	0 (0.0%)	3 (9.1%)	10 (30.3%)	20 (60.6%)	0 (0.0%)
Stories about others	0 (0.0%)	3 (9.1%)	16 (48.5%)	13 (39.4%)	1 (3.0%)
Suicide risk factors	0 (0.0%)	6 (18.2%)	14 (42.4%)	13 (39.4%)	0 (0.0%)
Evidence about restricting access to lethal means	0 (0.0%)	6 (18.2%)	16 (48.5%)	10 (30.3%)	1 (3.0%)
Impact of suicide	1 (3.0%)	6 (18.2%)	17 (51.5%)	9 (27.3%)	0 (0.0%)
Types of research studies	1 (3.0%)	6 (18.2%)	19 (57.6%)	6 (18.2%)	1 (3.0%)
	**Too long, f (%)**	**Just right, f (%)**	**Too short, f (%)**		**Skip/unsure, f (%)**
Web tool length	9 (27.3%)	21 (63.6%)	1 (3.0%)		2 (6.1%)
	**Too much, f (%)**	**Just right, f (%)**	**Too little, f (%)**		**Skip/unsure, f (%)**
Amount of information	7 (21.2%)	21 (63.6%)	5 (15.2%)		0 (0%)
	**Slanted toward in home options, f (%)**	**Balanced, f (%)**	**Slanted toward out of home options, f (%)**		**Skip/unsure, f (%)**
Balance of storage options	1 (3.0%)	30 (90.9%)	2 (6.1%)		0 (0%)
	**Yes, f (%)**	**No, f (%)**			**Skip/unsure, f (%)**
Enough information	27 (81.8%)	5 (15.2%)			0 (0%)

*f = frequency*.

* *Participants asked to rate what you think about the way the information was presented on each content area using a 4-point scale: “Poor” = 1, “Fair” = 2, “Good” = 3, and “Excellent” = 4. The ordering of the table is designed to highlight items that were weighted most heavily in development process. To view the original Acceptability Questionnaire, see Supplementary Figure S1*.

Qualitative data supporting acceptability and highlighting directions for possible improvements expand on these quantitative ratings (Table 4). As clear from the stakeholder comments shown in Table 4, length remained an issue for some stakeholders, and for some clinicians, the “nudging” approach was viewed as not as strong as the approach they took clinically.

**Table 4:** Qualitative feedback on “Lock and Protect” decision aid from parent and clinician stakeholders.

**Theme**	**Quotation**
**Presentation**	
Sample parent quote	“I love the presentation. Great, big fonts, pictorials, easy to understand language.”
Sample clinician quote	“I thought it was really well-balanced. It was pretty comprehensive, gave a lot of different options.”
**Ease of understanding/use**	
Sample parent quote	“I liked the user interface of the tool…I have more control. It's not one-size fits-all, like it's more personalized.”
Sample clinician quote	“It gives a parent very practical steps to follow and it isn't overwhelming.”
**Unique benefit**	
Sample parent quote	“It gave me suggestions that I hadn't considered before.”
Sample clinician quote	“It could be done regardless of disposition, like regardless of whether I'm advising the parent that we're going to admit or discharge because anyone who's admitted, eventually gets discharged.”
**Tone of tool**	
Sample parent quote	“These are not easy things for people to dialogue about and it seemed to have a very, I call it a soft, user-friendly feel for topics that are really hard for people to address.”
Sample clinician quote	“I think it kind of helps them understand that this isn't anything that they've done, this is something that your child is going through.”
**Areas for improvement**	
Sample parent quote	“I thought it was a little long but understandably so.”
Sample clinician quote	“I think if they got this tool after meeting with me, I think it could send mixed signals because it's a lot more forgiving than what I recommend.”

### Comparison With Earlier Iterations

Importantly, examination of responses across the 10 L&P iterations revealed relatively consistent feedback that L&P would be “useful if you were making a decision about reducing access to means for youth at risk of suicide.” Indeed, once the medication section and branching logic to reduce DA length were added to the firearms iteration, all participants rated the DA as useful for reducing lethal means access. Prior to that point when participants were shown only the firearms iteration or the longer iteration without branching, a small minority (3/52, 5.8%) rated the DA as not useful.

### Development Themes

A consistent stakeholder theme across iterations was length, resulting in efforts to balance coverage of key dangers and strategies for enhancing youth safety with the need to create a user-friendly acceptable tool. Branching logic allowed respondents to skip sections based on initial responses, which helped reduce length from 48 pages in the longest iteration to a final DA ranging from 10 to 19 pages. Parents reporting access to firearms viewed five additional pages, those reporting access to medicines viewed three more pages, and parents indicating that the child was left alone unsupervised saw one additional page. Additionally, parents had the option of viewing frequently asked questions and additional information on crisis resources. Participants also varied considerably in their opinions regarding the use of cameras and other remote monitoring options. Although no stakeholders reported that these options shouldn't be covered in the DA, and some parents used the approach; stakeholders often expressed strong feelings about the value of remote monitoring, and some expressed strong concerns that remote monitoring strategies could adversely affect the parent-child relationship (e.g., conflict, feelings of mistrust) further indicating that they would not use this method.

### Workflow

Parent and clinician feedback was consistent in indicating a need to consider both the emotional state of parents whose children are seen for emergency suicide-risk evaluations, and a preference for presenting the DA after parents met the clinician and conducted some risk evaluation. Integration of the DA within safety and/or discharge planning was described as optimal across stakeholder groups. All of the stakeholders from the workflow interviews (100%) reported that the DA could be feasibly integrated in the ED and noted the utility of this tool even in challenging environments when paired with provider guidance.

## Discussion

This manuscript describes our final iteration of the L&P DA, a parent/caregiver-facing web-based DA which offers a tool for completing lethal means counseling, a key component of suicide prevention care (5). Such low-cost resources offer opportunities to augment clinician-provided care, reduce required clinician time, and overcome system-level barriers, such as limited staffing and mental health clinician access. Stakeholder feedback, however, underscored the importance of delivery with a “human touch” (21), after some clinician contact/evaluation. Parents and clinicians further noted the value of offering the DA after families had a chance to process the suicidal episode and the focus was on discharge and safety planning. This is consistent with research indicating that digital interventions that included some contact with a clinician or individual acting as a coach resulted in stronger use and benefits, compared to digital interventions alone without such support (30).

Using a behavioral economics model, L&P presents information and gently “nudges” parents to identify options for (1) limiting access to firearms and other potentially lethal methods, and (2) providing protective and supportive supervision to prevent suicide attempts with methods that cannot realistically be eliminated. Consistent with the definition of nudges as features that attract the viewer's attention and increase the probability that they will freely make particular decisions and behave in specific ways that are in their self-declared best interests (31), L&P guides parents in (1) considering potentially lethal and dangerous self-harm methods within their homes, as well as times when youths may be alone with no one to protect them; (2) considering options for increasing safety (e.g., elimination, storage, supportive protective monitoring); and (3) “nudges” parents to identify and use self-selected options that parents believe will best achieve the goal of increasing safety. Further, the DA utilizes framing strategies to achieve this goal (31). When providing psychoeducation on means safety, and based on user feedback, we used language emphasizing the temporary nature of means removal and deliberately avoided the phrase “restriction,” especially around firearms. Stakeholder Feedback was positive regarding our approach of referencing the concept of “baby-proofing the home” and “nudging” toward recognition that, when a child is suicidal, protective action is similarly needed. Although most DAs are grounded in the idea of equipoise among available options (27), evidence on the potential life-saving impact of reducing access to lethal means support this “nudging” toward protective action (5, 32).

User feedback underscored variation in perceptions of necessary and acceptable protective actions. The greatest controversy involved remote monitoring (e.g., security cameras, baby-monitors). Some parents thought this could be useful, and a small number used cameras and monitoring of doors. Others viewed this as a sign of mistrust that would cause tension in the parent-child relationship and might miss risk behavior, thereby causing a false sense of security. The need for remote monitoring may also indicate the need for a more restrictive care setting. Given our mixed feedback, increasing use of remote monitoring generally, and use of CCTV and video in population-level automated detection systems for early intervention (e.g., at bridges to catch people before they jump) (33), we decided to retain this option and list raised concerns.

A key development decision involved whether to focus tightly on firearms, which would yield a shorter tool focusing on a highly lethal method. Alternatively, a broader focus allows greater reach to more patients, enhancing feasibility and potential suicide prevention value. Indeed, the adult L2L DA was expanded to address medication storage and translated to Spanish (20, 21). However, this broader focus lengthens the DA with potential for reducing user acceptability. There is also a possibility that limiting access to less lethal methods could lead some youth to substitute methods with greater lethality (5). Future work is needed to determine whether the broader focus of L&P has advantages from a clinical and service use perspective.

This is an initial development study that has limitations. Due to the high lethality of firearms as a suicide attempt method, our recruitment strategy emphasized firearm owners, who tend to be disproportionately White and non-Hispanic (34). This may have contributed to the relatively small number of ethnic and racial minorities in the sample. Future work is needed to assess the generalizability of study findings and acceptability and response to L&P among diverse stakeholders. L&P was developed and evaluated in English, a Spanish translation is in development. While there was consistency in stakeholder feedback, the sample of parents reviewing the final L&P iteration was also relatively small, and data were limited to acceptability and perceived value of the DA. Future trials are needed to evaluate whether L&P leads to behavioral changes that enhance youth safety, and to determine acceptability and impact among a larger, more diverse, and representative sample. While this study focused on the ED and emergency care, future research might explore the acceptability and value of L&P in other settings beyond the ED. Indeed, several stakeholders advocated for offering the DA to diverse service settings, including mental health and primary care services, which are especially relevant for preventing highly lethal suicide attempts (such as by firearm) that may result in death on the first attempt (35).

In conclusion, to our knowledge, L&P is the first web-based DA developed specifically for lethal means counseling in parents of suicidal youth. This digital tool can augment clinician-delivered services and provides a user-friendly approach for enhancing lethal means safety, a suicide prevention strategy with demonstrated effectiveness. Because the low rate of lethal means counseling as part of routine ED care may be due to time constraints and the challenges of ED care (18), the availability of a digital self-administered DA provides an easy to use protocol for guiding parents in assessing potential dangers for their child and taking actions to enhance means safety. Parents can review the information and share the summary page with their clinician, with clinicians briefly reviewing, reinforcing actions to enhance youth safety, and incorporating within the treatment plan. Recognizing the challenges of emergency care, L&P was developed as a self-guided approach that offers opportunities to address means safety with minimal burden on providers. Thus, we aimed to create a user-friendly tool that could be used to increase the likelihood that families will receive lethal means counseling when needed. Indeed, results of our clinician workflow interviews indicated that clinicians viewed L&P as a useful clinical tool that can be integrated within the ED workflow. The DA focus on parents is consistent with accumulating research demonstrating that interventions with parents and families are associated with increased treatment benefits among suicidal and self-harming youth, compared to individual therapy alone (36–38). Rigorous controlled trials are needed to clarify the value of L&P for motivating parents to act to reduce access to dangerous suicide attempt methods, strengthen protective actions, and reduce the risk of future fatal and non-fatal suicide attempts.

## Data Availability Statement

The raw data supporting the conclusions of this article will be made available by the authors, without undue reservation.

## Ethics Statement

The studies involving human participants were reviewed and approved by UCLA Institutional Review Boards, Office of Human Research Protection Program, IRB#17-001315. Written informed consent for participation was not required for this study in accordance with the national legislation and the institutional requirements.

## Author Contributions

JA, MB, and DG developed the idea and methodology. SE, LZ, and CV performed the data collection. JA, MB, DG, LZ, CV, and AT contributed to method implementation and analysis. JA and DG provided supervision. JA, DG, SE, LZ, and CV were involved in project administration. All authors contributed to the manuscript, read, and approved the submitted version.

## Funding

This work was partially supported by: U79SM080041 awarded by the Department of Health and Human Services, Substance Abuse and Mental Health Services Administration (SAMHSA, Asarnow and Goldston, Co-PIs); and NIMH R34MH113539 (Betz PI).

## Author Disclaimer

Points of view in this paper are those of the authors and do not necessarily represent the official positions or policies of the listed funding agencies.

## Conflict of Interest

JA has received grant, research, or other support from the National Institute of Mental Health (NIMH), the American Foundation for Suicide Prevention, the Substance Abuse and Mental Health Services Administration (SAMHSA), the American Psychological Foundation, the Society of Clinical Child and Adolescent Psychology (Division 53 of the APA), and the Association for Child and Adolescent Mental Health. She has served as a consultant on quality improvement for depression and suicide/self-harm prevention, serves on the Scientific Council of the American Foundation for Suicide Prevention, and the Scientific Advisory Board of the Klingenstein Third Generation Foundation. DG has received grant, research, or other support from NIMH, SAMHSA, the National Institute on Alcohol Abuse and Alcoholism, and the U.S. Department of Defense. The remaining authors declare that the research was conducted in the absence of any commercial or financial relationships that could be construed as a potential conflict of interest.

## Publisher's Note

All claims expressed in this article are solely those of the authors and do not necessarily represent those of their affiliated organizations, or those of the publisher, the editors and the reviewers. Any product that may be evaluated in this article, or claim that may be made by its manufacturer, is not guaranteed or endorsed by the publisher.
