# Supplementary material for: “Lock and Protect”: Development of a Digital Decision Aid to Support Lethal Means Counseling in Parents of Suicidal Youth
Source: Front Psychiatry. 2021 Oct 6;12:736236. doi: 10.3389/fpsyt.2021.736236 (PMC8528190; doi:10.3389/fpsyt.2021.736236)
Supplement: Supplementary file 1 [file Table_1.DOCX]

| **Table S1.** Summary of “Lock and Protect” iterations, testing round results, and development decisions. | | | |
| --- | --- | --- | --- |
| Iteration | DA Shown | Themes for Change | Decision |
| 0 | L2L, Adult and firearms focused | Needs adaptation for parents* of youth | Explore adaptation for parents |
| 1 | Introduction targeted to parents, with L2L Firearms. | Confirmed need to create DA specifically for parents of youth with elevated suicide risk | Create separate DA for parents of youth |
| 2 | Parent DA #1, firearms focused | Unclear what term to use to refer to the youth (your child vs. kid) | Used child to refer to your child, kid to refer to youth in general |
| 3 | Parent DA#2., firearms focused | Too long, need to address usual questions and barriers to change | Emphasized parent role in protection  Added FAQ |
| 4 | Parent DA#3, firearms focused | Shorten, suicide attempts by overdose and other methods are not addressed | Need to consider other suicide attempt methods for usability |
| 5 | Medication Version #1 + Other Dangerous Potential Means | Other high lethality methods cannot realistically be restricted, more attention needed to how parents could protect through supervision | Increased focus on other high lethality suicide attempt methods and protective supervision |
| 6 | Medication Version #2 | Consideration of medications and other high lethality means useful and important | Combine firearms and medications in single DA |
| 7 | Combined Firearms & Medication Version #1 | Too long | Use branching algorithms to shorten |
| 8 | Branching based on method presence | Too long. Acceptable and  useful for addressing lethal means | Refine branching to reduce length.  Need clinician feedback on workflow |
| 9 | Programmed with branching algorithms | Workflow interviews with providers. Useful tool to augment provider time, more detail on options for safety monitoring/supervision when parent not available should be considered, best to include decision aid after initial risk assessment as part of safety and discharge planning | Expanded information on options for safety monitoring/supervision when parent not available. Ready for final test round with parents |
| 10 | Programmed with branching algorithm | Final test round with parents. Useful and acceptable tool. Mixed feedback on security camera/baby monitor option | Modified presentation of security camera/baby monitor option- revised and presented again to participating parents. Ready for testing. |
| *”Parents” refers to parents or caregivers | | | |
