# Supplementary material for: “Lock and Protect”: Development of a Digital Decision Aid to Support Lethal Means Counseling in Parents of Suicidal Youth
Source: Front Psychiatry. 2021 Oct 6;12:736236. doi: 10.3389/fpsyt.2021.736236 (PMC8528190; doi:10.3389/fpsyt.2021.736236)
Supplement: Supplementary file 2 [file Image_1.PDF]

Figure S1. “Lock and Protect” Decision Aid Acceptability Questionnaire.

1. Please rate each section, by reporting 'poor', 'fair', 'good', or 'excellent' to show what you think about the **way the information was presented** on:

	POOR (1)	FAIR (2)	GOOD (3)	EXCELLENT (4)
Impact of Suicide	○	○	○	○
Risk Factors	○	○	○	○
Types of Research Studies	○	○	○	○
Role of Lethal Means (e.g., Firearms, Medications)	○	○	○	○
Evidence about Reducing Access to Lethal Means	○	○	○	○
Options for Reducing Access to Lethal Means	○	○	○	○
Stories about Others	○	○	○	○

2. The **length** of presentation was (*check one*):
   ○ Too long
   ○ Just right
   ○ Too short

3. The **amount** of information was (*check one*):
   ○ Too much information
   ○ Just right
   ○ Too little information

4. I found the presentation (*check one*):
   ○ Slanted towards options in the home (like safes)
   ○ Slanted towards options outside of the home (like storage elsewhere)
   ○ Balanced

5. Would you have found this decision aid useful if you were making a decision about reducing access to means for youth at risk of suicide?
   ○ Yes
   ○ No
   Comments:

6. Do you think we included enough information to help a person decide on ways to reduce access to lethal means, like firearms, at home?
   ○ Yes
   ○ No
   Comments:

7. What did you like about the decision aid?

8. What **suggestions** do you have to improve the decision aid?
